# The Role of Affective Control in Emotion Regulation During Adolescence

**DOI:** 10.1037/emo0000695

**Published:** 2020-02

**Authors:** Susanne Schweizer, Ian H. Gotlib, Sarah-Jayne Blakemore

**Affiliations:** 1 Developmental Cognitive Neuroscience Group, Institute of Cognitive Neuroscience, University College London; 2 Department of Psychology, Stanford University; 3 Developmental Cognitive Neuroscience Group, Institute of Cognitive Neuroscience, University College London

**Keywords:** emotion regulation, cognitive control, development, executive functions, affective control

## Abstract

In this review, we evaluate evidence for the hypothesis that developmental changes in emotion regulation tendencies during adolescence depend on the maturation of affective control. Affective control refers to the application of cognitive control to affective contexts, that is, the capacity to attend and respond to goal-relevant affective information, while inhibiting attention and responses toward distracting affective information. The evidence suggests that affective control develops throughout adolescence into adulthood. However, the developmental trajectory appears not to be uniform across different facets of affective control. In particular, the capacity to inhibit attention and responses to distracting affective information may be reduced during adolescence relative to childhood and adulthood. Focusing on the association between affective control and emotion regulation development in adolescence, the research reviewed supports the notion of affective control as a cognitive building block of successful emotion regulation. Good affective control appears related to fewer ruminative tendencies in adolescence as well as more frequent and successful reappraisal in older adolescents. Lower use of habitual suppression, itself a type of affective inhibition, shows an association with updating capacity. We conclude by discussing the implications of these findings for mental health and the potential mental health benefits associated with improving affective control.

Adolescents experience greater affective fluctuations, higher levels of negative mood, and are more sensitive to the experience of positive, rewarding experiences than are adults and children (Riediger, Wrzus, Schmiedek, Wagner, & Lindenberger, 2011; Spear, 2011). A subset of adolescents find these affective experiences challenging to regulate. Emotional dysregulation has been prospectively associated with poor mental health in adolescence (e.g., McLaughlin, Hatzenbuehler, Mennin, & Nolen-Hoeksema, 2011). Understanding how emotion regulation develops during adolescence is therefore important when considering targets for prevention and early intervention for mental health problems. Here, we build on cognitive neuroscience perspectives of emotion regulation, which argue that cognitive control is central to successful emotion regulation (Ochsner & Gross, 2005). Cognitive control refers to the capacity to attend and respond to goal-relevant information and inhibit attention and responses to distracting, goal-irrelevant inputs (Braver, 2012). The maturation of different components of cognitive control has been posited to drive developmental changes in emotion regulation tendencies during adolescence (Luciana, 2013). We propose that it is the development of affective control, the application of cognitive control in affective contexts, that is central to the development of emotion regulation. Understanding the developmental trajectory of the association between affective control and various types of emotion regulation tendencies provides insights into potential windows of opportunity for improving adolescent emotion regulation.

The first part of this review defines emotion regulation before focusing on studies exploring developmental changes in affective control in adolescence (10–24 years; Sawyer, Azzopardi, Wickremarathne, & Patton, 2018). In the second part, we explore how affective control relates to the adolescent development of different emotion regulation tendencies and conclude by discussing the implications for mental health.

## Emotion Regulation

Following Gross’ extended process model of emotion regulation (Gross, 2015; see also McRae & Gross, 2020 in this issue), we define emotion regulation as the modification of emotional states through a three-stage process: identification, selection, and implementation. In the identification stage, emotions are perceived and valued as sufficiently negative/positive to require regulation. In the second selection stage, potential emotion regulation strategies are represented and evaluated against available resources, leading to the goal representation of using the selected strategy. In the final implementation stage, the contextual factors are perceived and then evaluated to determine the most promising approach to implementation of the selected strategy.

Like all regulatory functions, our capacity to regulate our emotions improves from early life to adulthood (Cole, Martin, & Dennis, 2004). Improvements are observed across all three stages of the emotion regulation process, though developmental trajectories are not uniformly linear. For example, emotional differentiation, which is critical to the identification stage, decreases from childhood to adolescence, then improves again throughout adolescence and stabilizes in adulthood (Nook, Sasse, Lambert, McLaughlin, & Somerville, 2018). Increased habitual use (Zimmermann & Iwanski, 2014) and proficient implementation (McRae, Gross, et al., 2012) of cognitively demanding strategies, such as cognitive reappraisal, appears to increase more linearly from late childhood to adulthood. At the neural level, age-related improvements in emotion regulation are proposed to depend on strengthened connectivity between prefrontal brain regions (implicated in cognitive control) and emotion and reward processing regions, including the amygdala and striatum (for a review, see Aldao, Gee, De Los Reyes, & Seager, 2016). These emotion regulation and processing regions are critical in the perception and valuation of emotions at each stage of emotion regulation.

The habitual use of more complex strategies, as well as an increased ability to switch between strategies in response to changing situational demands (Aldao, Sheppes, & Gross, 2015), are postulated to emerge as a function of improved cognitive control capacity. We argue that changes in emotion regulation are, in particular, related to developing affective control. We next review evidence for adolescent development of affective control: the capacity to flexibly engage and disengage with affective information depending on changing goal demands.

## Developing Affective Control

Developmental researchers have investigated “hot” and “cool” cognitive control, often comparing age-related performance differences using different tasks (e.g., hot: Iowa gambling task vs. cool: classic Stroop task; Welsh & Peterson, 2014). Using tasks that vary both in the required cognitive processes (e.g., decision making vs. inhibition) and in their affective significance (hot/affective vs. cool/neutral) introduces the possibility that variations in developmental trajectories are accounted for by age-related differences in task-specific factors other than valence (Welsh & Peterson, 2014). Therefore, we focus here on studies that compare developmental changes on the same cognitive control task performed in hot versus cool contexts (i.e., using affective vs. neutral material). This allows us to explore the relative contribution of affective control, compared to cool cognitive control exerted in comparatively neutral contexts.

**Figure 1 fig1:**
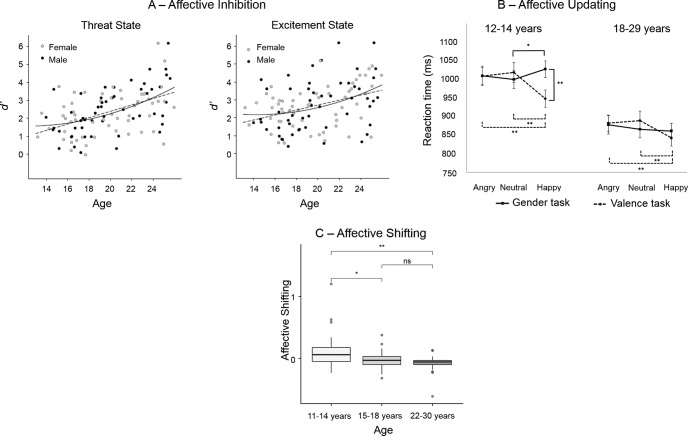
Development of the three facets of affective control. The three panels show different facets of affective control (task performance/reaction time [RT] is always contrasted with performance in neutral contexts). (A) Age-related improvements from adolescence to adulthood in affective inhibition measured as *d*′ (ratio of hits to misses) on a go/no-go task performed in experimentally induced affective states of threat (anticipation of noxious noise) and excitement (anticipation of monetary reward) compared to a neutral state. The left panel shows a linear improvement in inhibition performance with age in the neutral state (i.e., cool cognitive control). The middle and right panels show a quadratic association between task performance and age when experiencing threat and excitement (i.e., affective control), respectively. From “When Is an Adolescent an Adult? Assessing Cognitive Control in Emotional and Nonemotional Contexts,” by A. O. Cohen, K. Breiner, L. Steinberg, R. J. Bonnie, E. S. Scott, K. A. Taylor-Thompson, . . . B. J. Casey, 2016, *Psychological Science, 27,* p. 557. Copyright 2016 by SAGE. Reprinted with permission. (B) An updating task with two conditions, both of which require affective control. Whereas in the valence task affective control is required to update affective material, in the gender task individuals are required to inhibit affective task-irrelevant features in order to attend to their nonaffective features (gender task). The gender task shows age-specific slowing in affective updating of task-irrelevant happy cues on an *n*-back task. This is demonstrated in the left panel, which shows adolescents’ slowed RTs to happy faces compared to neutral and angry faces when they are task-irrelevant (gender task/solid line). In the right panel, adults’ RTs to affective stimuli are unaffected by the valence of the stimuli in the gender task. In contrast, there are no age-related effects on affective updating when the memoranda are affective. That is, updating is fastest for positive cues in the valence task (stippled line) for both adults and adolescents. From “The Power of a Smile: Stronger Working Memory Effects for Happy Faces in Adolescents Compared to Adults,” by S. Cromheeke and S. C. Mueller, 2016, *Cognition and Emotion, 30,* p. 294. Copyright 2016 by Taylor & Francis. Reprinted with permission. (C) Better affective shifting (measured as the proportional difference in errors for the affective relative to neutral condition) performance in early adolescence than later in development is shown. Unlike inhibition and updating, these results suggest greater affective shifting capacity in early adolescence compared to later in development when performance is no longer affected by the valence of the stimuli. From “Age-Related Differences in Affective Control and Its Association With Mental Health Difficulties,” by S. Schweizer, J. Parker, J. T. Leung, C. Griffin, and S.-J. Blakemore, 2019, *Development and Psychopathology* (in the public domain).

The development of affective control has been investigated with tasks measuring inhibitory control in affective versus neutral contexts, typically with versions of the affective go/no-go (e.g., Tottenham, Hare, & Casey, 2011) or emotional Stroop (e.g., Lagattuta, Sayfan, & Monsour, 2011) tasks. These studies have generally found a linear age-related reduction in interference from affective stimuli, mirroring the developmental pattern observed for the inhibition of neutral stimuli, yet the time course appears more protracted (Tottenham et al., 2011). However, some studies have found a quadratic effect of development. For example, Somerville, Hare, and Casey (2011) showed reduced response inhibition to happy, but not neutral, faces in adolescents compared to both children and adults. Cohen et al. (2016) replicated the finding of greater interference from positive faces in younger adolescents (13–17 years) than in older adolescents (18–21 years; the original paper referred to this group as young adults) and adults (22–25 years), while showing a more linear improvement of inhibitory control of responses to negative and neutral faces. That is, inhibition of negative faces was lowest in the young adolescent group, intermediate in the older adolescent group and highest in the adult group. Similarly, older adolescents did not differ with either young adolescents or adults in reaction time to neutral faces, but inhibition in response to neutral faces was slowed in young adolescents relative to adults. Compared with adults, adolescents also showed a greater interference effect of positive and negative emotional states (Figure 1A), induced by the anticipation of unpredictable monetary rewards and noxious noise, respectively. Furthermore, increasing age was associated with stronger coupling between the prefrontal cortex and task-relevant brain regions (including the dorsal anterior cingulate; Cohen et al., 2016).[Fig-anchor fig1]

Typical development of affective control has also been measured with updating (e.g., Cromheeke & Mueller, 2016; Mueller, Cromheeke, Siugzdaite, & Nicolas Boehler, 2017) and shifting tasks (e.g., Mărcuş, Stanciu, MacLeod, Liebregts, & Visu-Petra, 2016; Schweizer, Parker, Leung, Griffin, & Blakemore, 2019). For example, a study compared performance in adults (18–29 years) and young adolescents (12–14 years) on an *n*-back task that required updating of emotional faces’ gender in the neutral condition versus updating the faces’ emotional expressions in the affective condition (Cromheeke & Mueller, 2016; Figure 1B). Adolescents’ updating capacity was similar to that of adults when updating emotional expressions, but was reduced when updating the gender of happy faces (Cromheeke & Mueller, 2016). Attention to the affective information needed to be inhibited in the gender condition; further supporting reduced affective inhibition during adolescence. This contrasts with affective shifting, where we showed that, while young adolescents (11–14 years) had overall lower shifting performance than did older age groups, their performance was superior to that of older groups in an affective compared to a neutral task context (Figure 1C; Schweizer, Parker, et al., 2019).

Taken together, these studies suggest that affective control does not develop as a unitary construct. Instead, preliminary evidence suggests that affective control, particularly, inhibition of affective information, is reduced during adolescence, but less so affective shifting and updating. Despite apparently different developmental trajectories, poor affective control during adolescence across all three facets is associated with more mental health problems (Kilford et al., 2015; Ladouceur et al., 2013; Schweizer, Parker, et al., 2019). Impairments in affective control may help to explain the increased negative affect and greater affective fluctuations during this period of life (Aldao et al., 2016; Riediger et al., 2011).

## Affective Control and Emotion Regulation Across Development

Although longitudinal research is currently lacking, in this section we review whether affective control is associated with emotion regulation tendencies in adolescence. Specifically, we focus on rumination, suppression, and reappraisal, where excessive rumination reflects difficulties with the inhibition of repetitive, perseverative negative thinking (Gotlib & Joormann, 2010); emotion suppression involves affective inhibition of affective states or expressions (Chervonsky & Hunt, 2017); and reappraisal requires individuals to shift and update their interpretations of affective experiences, thoughts, and events (Aldao, Nolen-Hoeksema, & Schweizer, 2010).

### Rumination

A breakdown in cognitive control has been proposed to lie at the core of maladaptive rumination, according to meta-analytic evidence from studies conducted primarily in adults (Yang, Cao, Shields, Teng, & Liu, 2017). In contrast, habitual rumination in young adolescents (12–13 years) was unrelated to cool cognitive control cross-sectionally, and cognitive control did not predict habitual use of rumination 15 months later Connolly et al. (2014). The lack of a prospective association between cognitive control and rumination reported by Connolly et al. (2014) may be accounted for by the fact that individual differences in affective control, but not cognitive control applied in relatively neutral contexts, predict habitual use of rumination. This hypothesis is supported by research showing that affective control deficits, operationalized as poorer inhibition of attention toward affective faces on a dot-probe task, were associated with a stronger tendency to ruminate in young adolescents (11–14 years) with a history of childhood maltreatment (Romens & Pollak, 2012). Similarly, the inhibition of responses to affective relative to neutral faces on a go/no-go task was reduced in older children and adolescents (9–16 years) who reported high levels of habitual rumination compared to low ruminators (Hilt, Leitzke, & Pollak, 2014, 2017).

### Suppression

Habitual use of suppression in adolescents and adults is associated with poor mental health and impaired social functioning (Chervonsky & Hunt, 2017, 2019). This is in contrast with early childhood when suppression ability is a marker of good emotion regulation (Cole & Jacobs, 2018). Age-related differences in the relative adaptiveness of suppression appear to develop in tandem with a shift in the underlying cognitive control component on which successful suppression depends. Specifically, in 4–6 year-olds, parental ratings of greater habitual emotional suppression were moderately associated with better inhibitory control of behavioral responses (Carlson & Wang, 2007). In contrast, in adolescents less habitual suppression was associated with greater self-reported updating capacity, but not with inhibitory control (Lantrip, Isquith, Koven, Welsh, & Roth, 2016). This association fits with the finding that attention suppression to salient visual distractors is dependent on working memory capacity in adults (Gaspar, Christie, Prime, Jolicœur, & McDonald, 2016). However, we should note that these findings are limited to cool cognitive control. There is currently little research examining the association between affective control and habitual suppression in adolescence. Given the association between suppression and negative mental health outcomes in youth (Schäfer, Naumann, Holmes, Tuschen-Caffier, & Samson, 2017), it is critical that we gain a better understanding of affective control in suppression.

### Reappraisal

Reappraisal refers to the capacity to downregulate affective experiences by cognitively reinterpreting the meaning of the emotion-eliciting event. Across a wide range of mental health problems, individuals report low levels of habitual reappraisal (Aldao et al., 2010). In fact, research demonstrating a shared underlying neural architecture between successful reappraisal and cognitive control was fundamental to the formulation of neuroscientifically informed theories of emotion regulation (Ochsner & Gross, 2005). Building on these theories, evidence from studies in adults shows that affective control, in particular affective updating, is associated with the habitual use of reappraisal (e.g., Vanderhasselt, Baeken, Van Schuerbeek, Luypaert, & De Raedt, 2013). Like suppression, there is less research examining the association between affective control and reappraisal in adolescence. Therefore, we draw on both studies of cool cognitive control and affective control in this paragraph. Research exploring developmental differences in reappraisal has shown that, compared with adults, adolescents show less efficient recruitment of cognitive control regions to downregulate amygdala reactivity during reappraisal (Silvers, Shu, Hubbard, Weber, & Ochsner, 2015). Another study showed that older adolescents and emerging adults (*M*_age_ = 19 years), who experienced higher levels of interference from negative material on a working memory updating task, demonstrated less efficient increases in positive affect following reappraisal in everyday life (Pe et al., 2013). While the association between reappraisal and affective control in younger adolescents remains underexplored, cool cognitive control was associated with greater reappraisal tendencies across adolescence (12–18 years; Lantrip et al., 2016). McRae, Gross, et al. (2012) also observed that, in adults, behavioral measures of cool cognitive control were associated with more efficient use of reappraisal. Of note is that the association was specific to the updating working memory (Lantrip et al., 2016; McRae, Jacobs, Ray, John, & Gross, 2012) and shifting (McRae, Jacobs, et al., 2012) components of cognitive control, and not the inhibition component (Lantrip et al., 2016; McRae, Jacobs, et al., 2012).

In combination, the cross-sectional evidence shows that better affective control is associated with less habitual rumination as well as more frequent and more successful use of reappraisal. Suppression, itself a type of affective inhibition, is most strongly associated with updating capacity. A point to note when considering the association between habitual emotion regulation and affective control is that the development of increasingly robust emotion regulation tendencies across adolescence may simultaneously increase the levels of affective control required to override these tendencies in response to affective (but not neutral) information presented in control tasks. For example, high ruminators not only have lower levels of affective control capacity, but the tendency to ruminate may further increase the affective significance of negative material and consequently augment its interference potential.

## Affective Control, Emotion Regulation, and Mental Health

The literature reviewed supports our proposal of affective control as a cognitive building block of successful emotion regulation. This conclusion highlights the need for longitudinal research exploring the codevelopment of affective control and emotion regulation across adolescence. The findings from studies using different affective control paradigms, which assess affective inhibition, updating and shifting, suggest that affective control is not a unitary construct, and that each component may show a different developmental trajectory.

This review has focused on the typical development of affective control and emotion regulation. A major motivation to understand the typical development of the cognitive and neural architecture underlying emotion regulation is to study individual differences, as this might shed light on the development of poor emotion regulation tendencies, which constitute a core causal pathway to mental health problems in adolescence (Aldao et al., 2016; McLaughlin et al., 2011). Affective control deficits, then, are expected to be associated with mental health problems. Results from a large-scale meta-analysis, primarily based on adult data, showed that poor affective control indeed differentiated between psychologically healthy individuals and individuals experiencing a wide range of mental health problems including depression, anxiety disorders, schizophrenia, and eating disorders (Schweizer, Satpute, et al., in press). Evidence from adolescent samples supports the association between the different facets of affective control and mental health outcomes. Compared to their typically developing and not at-risk peers, adolescents with, and at risk, for mental health problems show altered affective control capacity across all facets of affective control: inhibition (Kilford et al., 2015), updating (Ladouceur et al., 2013), and shifting (Mărcuş et al., 2016). In adolescence, affective control was further shown to account for variance in the association between emotion regulation and mental health (Schweizer, Parker, et al., 2019).

Training studies have shown in adolescents and young adults that affective control can be improved and that the gains in affective control are associated with improved emotion regulation and reduced mental health symptoms (e.g., Schweizer et al., 2017). The promising preliminary findings now require replication in larger-scale studies. Building this evidence is critical in order to open new avenues for prevention and early intervention strategies that target emotional dysregulation in adolescence.

## Future Research

The current review predominantly focused on the association between affective control and emotion regulation tendencies. That is, habitual use of specific emotion regulation strategies. However, emotion regulation capacity, that is the ability to successfully implement situationally adaptive emotion regulation strategies, has also been shown to improve with age (McRae, Gross, et al., 2012) and should be similarly dependent on affective control development. Future work should examine the contribution of affective control to the development of successful emotion regulation implementation. Another gap revealed in the present review is that the relative contribution of each of the facets of affective control (i.e., inhibition, updating, shifting) compared to cool cognitive control is poorly understood and will require psychometric investigations. Improving our mechanistic understanding of their relative contributions is key to tailoring efficient interventions aimed at improving emotion regulation development in adolescence.

Please refer to the online supplemental materials for recommended additional readings on this topic.

## Supplementary Material

10.1037/emo0000695.supp
