# Supplementary material for: The Role of Affective Control in Emotion Regulation During Adolescence
Source: Emotion. 2020 Feb;20(1):80–6. doi: 10.1037/emo0000695 (PMC6975522; doi:10.1037/emo0000695)
Supplement: Supplementary file 1 [file EMO-2018-1204_Supplemental_Materials.docx]

**Recommendation for Additional Reading**

Ahmed, S. P., Bittencourt-Hewitt, A., & Sebastian, C. L. (2015). Neurocognitive bases of emotion regulation development in adolescence. *Developmental Cognitive Neuroscience, 15*, 11-25.

This review provides an excellent foundation on the neural correlates of the development of

emotion regulation and affective control.

Cohen, A. O., Breiner, K., Steinberg, L., Bonnie, R. J., Scott, E. S., Taylor-Thompson, K. A., … Casey, B. J. (2016). When is an adolescent an adult? Assessing cognitive control in emotional and nonemotional contexts. *Psychological Science*, *27*(4), 549–562.

The study provides an illustration of developmental difference in *affective inhibition*, with adolescents showing reduced inhibitory capacity across different types of affective contexts (affective faces, monetary rewards and threat).

Schweizer, S., Parker, J., Leung, J. T., Griffin, C., & Blakemore, S.-J. (In Press). Age-related differences in affective control and its association with mental health difficulties. *Development and Psychopathology*.

This study shows that poor *affective shifting* capacity is associated with greater emotion regulation and mental health difficulties. The predictive utility of affective shifting appears greatest in early adolescence compared to both later adolescence and adulthood.

Schweizer, S., Satpute, A. B., Atzil, S., Field, A. P., Hitchcock, C., Black, M., & Dalgleish, T. (In Press). The behavioral and neural effects of affective information on working memory performance: A pair of meta-analytic reviews. Psychological Bulletin.

This paper provides an overview of the literature on *affective updating*, showing that altered affective updating capacity appears to be a transdiagnostic marker of mental health problems.

Hilt, L. M., Leitzke, B. T., & Pollak, S. D. (2017). Can’t take my eyes off of you: Eye tracking reveals how ruminating young adolescents get stuck. *Journal of Clinical Child & Adolescent Psychology*, *46*(6), 858–867.

This study shows that poor affective updating is already associated with increased tendencies toward rumination in early adolescence.
